# Thiazolidinediones for nonalcoholic steatohepatitis: A meta-analysis of randomized clinical trials

**DOI:** 10.1097/MD.0000000000004947

**Published:** 2016-10-21

**Authors:** Lingling He, Xiaoli Liu, Lijia Wang, Zhiyun Yang

**Affiliations:** a The Department of Traditional Chinese Medicine, Beijing Ditan Hospital; b Collabrorative Innovation Center of Infectious Diseases (ZY), Capital Medical University, Chaoyang, Beijing, China.

**Keywords:** meta-analysis, nonalcoholic steatohepatitis, thiazolidinediones

## Abstract

The findings regarding the effects of thiazolidinediones (TZDs) in nonalcoholic steatohepatitis (NASH) patients have been inconsistent, and the assessment of different clinical variables for evaluating the effects of TZDs confound a direct comparison of the results of different randomized clinical trials (RCTs), especially with regard to lifestyle changes. In this paper, we performed a meta-analysis of randomized controlled trials to clarify the effects of TZD treatment with and without lifestyle changes on histological markers of NASH and clinical variables related to insulin resistance (IR), hyperlipidemia, and obesity. We searched the literature using the following MeSH terms: “nonalcoholic steatohepatitis,” “non-alcoholic steatohepatitis,” “thiazolidinedione,” “pioglitazone,” “rosiglitazone,” “randomized,” and “clinical trial.” Five eligible RCTs were selected, in which patients were treated with either pioglitazone or rosiglitazone, with or without lifestyle changes. We compared the effects of TZD treatment on hepatic fibrosis, lobular inflammation, IR improvement, fasting serum insulin, adiposity, and dyslipidemia between the various studies using fixed and random effects models, and heterogeneity in clinical outcomes was assessed. Significant improvement in hepatic fibrosis did not occur among the patients treated with TZDs alone or in those who underwent both lifestyle changes and TZD therapy. Lobular inflammation decreased in NASH patients who received TZD treatment and in those who underwent both TZD therapy and lifestyle changes. Although TZD treatment resulted in no significant improvement in IR, NASH patients who underwent both lifestyle changes and TZD therapy experienced a significantly greater reduction in their fasting insulin level than that observed in the control patients, whereas patients treated with TZDs alone did not. Although TZD-treated patients experienced significantly greater weight gain than the control patients, TZD treatment had no significant impact on body-mass index, percentage of body fat, or serum levels of cholesterol and triglyceride. Our findings indicate that additional variables should be assessed to obtain a more comprehensive evaluation of the effects of TZD treatment on IR and comorbidity risk factors in NASH patients, and suggest that including lifestyle changes and additional insulin-sensitizing agents in TZD regimens might improve the benefits of TZD therapy for NASH.

## 1 Introduction

Nonalcoholic steatohepatitis (NASH) is a histologically severe form of nonalcoholic fatty liver disease (NAFLD) that can progress to hepatic fibrosis and cirhosis.^[1]^ In most cases, the presence of >5% macrovesicular steatosis, inflammation, and hepatocellular ballooning or other hepatocyte necrosis, typically predominating in acinar zone 3, comprise the minimum criteria for the histological diagnosis of NASH.^[2]^ A number of different mechanisms in hepatocytes are known to contribute to fatty degeneration of the liver in the development of NAFLD, including increased lipogenesis, decreased lipid excretion, and reduced oxidation of free fatty acids (FFAs).^[3–8]^ Although the factors contributing to NASH pathogenesis and its progression to fibrosis and cirrhosis are less clear, lipid peroxidation in hepatocytes and proinflammatory cytokines and adipokines are thought to play important roles.^[3–8]^

In recent years, NASH has become the most prevalent form of liver disease in industrialized nations,^[9–11]^ and it is rapidly becoming the leading indicator for liver transplantation.^[4,12]^ The incidence of NASH in China has also increased recently.^[13]^ Increased risks of insulin resistance (IR), cardiovascular disease (CVD), and metabolic syndrome (MetS) are associated with NASH.^[14–18]^ The development of IR increases the serum level of FFAs, which increases hepatocyte uptake of FFAs, leading to mitochondrial β-oxidation overload, the net effect of which can contribute to hepatic steatosis and NAFLD progression.^[15,19]^ Hyperinsulinemia due to IR increases the levels of FFAs and triglycerides in hepatocytes by increasing glycolysis and decreasing the production of apolipoprotein B-100.^[19]^The link between hepatic steatosis and IR suggests the importance of using treatment regimens that have beneficial effects on both NASH and IR.

The thiazolidinediones (TZDs), pioglitazone and rosiglitazone, modulate insulin sensitivity in a variety of tissues via peroxisome proliferator activated receptor-γ signaling, and have been shown to improve blood glucose control, as well as NASH-related parameters in clinical studies.^[14,20–22]^ Diet control and exercise have also been shown to improve both IR and NASH when combined with TZD drug therapy in clinical trials.^[23,24]^ However, the findings regarding the effects of TZDs on NASH have been somewhat inconsistent, due to differences in intervention strategies, histological scoring, and reporting methods.^[25]^ In addition, the use of different assessment variables and reporting methods for evaluating the effects of TZDs on insulin sensitivity in NASH patients confound a direct comparison of the therapeutic effects of TZDs and lifestyle changes on IR. We performed a meta-analysis of randomized controlled trials (RCTs) to clarify the effects of TZD treatment with and without lifestyle changes on glycemic control, serum lipids, obesity-related characteristics, and NASH-related histological variables.

## 2 Methods

### 2.1 Data sources and search strategy

Our meta-analysis was performed according to the recommendations of the Cochrane Handbook,^[26]^ and this report was prepared according to the Preferred Reporting Items for Systematic Reviews and Meta-Analyses (PRISMA) guidelines.^[27]^ We searched the MEDLINE, EMBASE, EBSCO, Springer, Ovid, and Cochrane Library databases for published reports of RCTs published in English that evaluated the effects of TZDs on NASH clinical outcomes. The following keywords were used for the literature search: “nonalcoholic steatohepatitis,” “non-alcoholic steatohepatitis,” “thiazolidinedione,” “pioglitazone,” “rosiglitazone,” “randomized,” and “clinical trial.” We used a date range for our search ending in 2015. Our study was approved by the Research Ethics Committee at our institution.

### 2.2 Selection criteria and outcome assessment

Two reviewers (XL and LH) independently reviewed the full-text versions of all the articles retrieved in the literature search to identify eligible studies. Conflicts were resolved by a third reviewer (ZY). The following inclusion criteria were used for study selection: RCT in which a TZD was used to treat NASH, included a control or placebo group for comparison, patients received a diagnosis of NASH on the basis of histological examination of liver biopsy, and all patients were ≥18 years of age. The exclusion criteria were as follows: no randomization; patients were treated with a combination of TZDs and other drugs; and included patients diagnosed with NAFLD without NASH, other severe liver diseases, any malignancy, heart failure, or kidney failure. No limitations were made on the basis of sex or language. We analyzed the following primary outcomes: Histological response to treatment based on changes in steatosis grade and lobular inflammation score, change in IR based on the homeostasis model assessment (HOMA-IR),^[24]^ and fasting serum insulin level. Changes in the following patient characteristics and serum lipid levels were monitored to assess potential adverse effects of TZD treatment: body weight, body mass index (BMI), percentage body fat, total serum cholesterol, and serum triglyceride.

### 2.3 Quality assessment and data extraction

Quality assessment was based on the following domains: randomization, allocation concealment, and blinding of participants and outcome assessors. The following data were extracted by a single investigator (LW): general information, including the study title, authors, and publication date; study features, including study design, outcomes assessed, bias prevention, outcomes reported, management of withdrawals, and adverse effects reported; and treatment details, including TZD dosage, duration of treatment, and length of follow-up. Categorical variables were recorded as incidences, and continuous variables were recorded as the mean and standard deviation (SD) of changes from baseline.

### 2.4 Statistical analysis

All statistics were computed using the RevMan, version 5.3.5, program provided by the Cochrane Collaboration website (http://tech.cochrane.org/revman/download). Heterogeneity in the datasets was assessed on the basis of the *I* statistic, with *I*^*2*^ >50% indicating significant heterogeneity. Random and fixed effects regression models were used to evaluate the effects of treatment for comparisons in which significant heterogeneity in the dataset was or was not detected, respectively. The results of the random and fixed effects models with *P* < 0.05 were considered to represent statistically significant differences. The histological assessments were treated as dichotomous categorical variables, for which the treatment effects were evaluated on the basis of the odds ratio (OR) and 95% confidence interval (95% CI). The body characteristics and serum biochemical variables were treated as dichotomous continuous variables. The mean difference (MD) and 95% CI were calculated for continuous variables if the measurement scales were identical in the studies compared. If the measurement scales were not identical, the standardized MD (SMD) and 95% CI were calculated. Forest plots were constructed to present the comparisons of the treatment effects between the RCTs.

## 3 Results

### 3.1 Study selection

A total of 75 publications were initially retrieved. After reviewing the titles and abstracts of the retrieved articles, 62 were excluded, 5 of which were meta-analyses or reviews. Thirteen studies underwent full-text review, after which 8 studies were deemed ineligible because the treatments included drugs other than TZDs or because patients with NAFLD without NASH were included. The 5 remaining studies were selected for our meta-analysis.^[23,24,28–30]^

### 3.2 Study characteristics

The characteristics of these studies are summarized in Table 1. Three of the RCTs were multicenter studies,^[23,28,30]^ and 2 were single-center studies.^[24,29]^ These 5 RCTs included a total of 405 participants who ranged in age from 46 to 54 years, and were performed in the USA, France, England, and Turkey between 2006 and 2010. Randomization was performed in all of the studies. Double blinding was performed in 4 of the studies,^[23,28–30]^ whereas only blinding of the assessors was reported in the remaining study.^[24]^ Only 1 of the selected RCTs performed allocation concealment.^[29]^ All of the selected RCTs included patients treated with either pioglitazone^[23,28,30]^ or rosiglitazone.^[24,29]^ The effects of TZD treatment alone were compared with those of a placebo alone in 3 of the studies.^[28–30]^ In 2 of the RCTs, patients who received TZD treatment also implemented lifestyle changes, which consisted of diet control^[23]^ or diet control along with exercise,^[24]^ and the effects of the combination of TZD treatment and lifestyle changes were compared with those of the lifestyle changes with and without placebo, respectively. Histological response and various adverse effects were reported in all of the RCTs included in our meta-analysis.

**Table 1:**
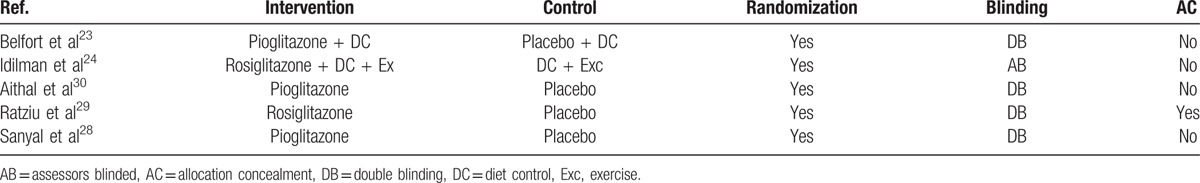
Characteristics of studies included in meta-analysis.

### 3.3 Effects of TZD treatment on fibrosis are unclear

**Figure 1:**
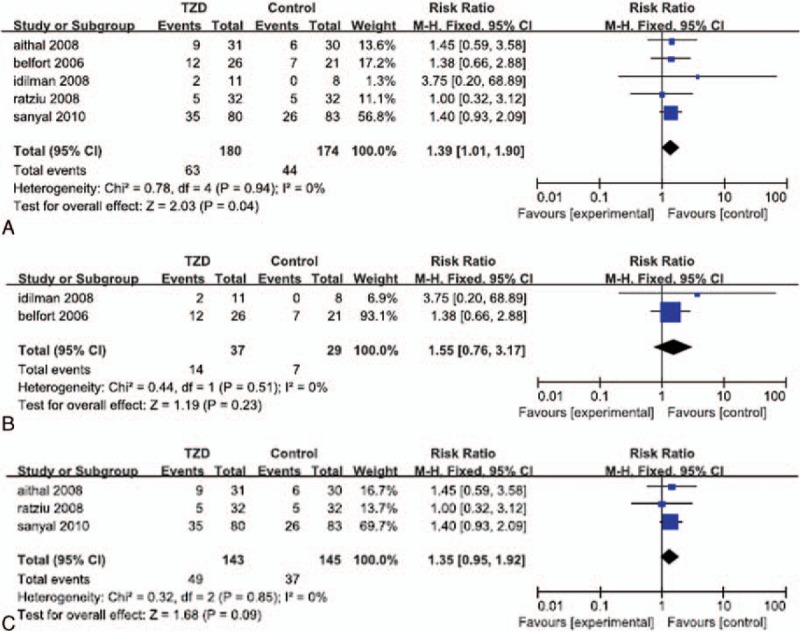
Improvement in hepatic fibrosis in patients treated with TZDs. (A) Overall analysis of improvement in fibrosis. (B) Subgroup analysis of improvement in fibrosis in patients who underwent lifestyle changes with or without TZD therapy. (C) Subgroup analysis of improvement in fibrosis of patients treated with TZDs alone.

Significant heterogeneity in the data regarding histological response was not detected (*I*^*2*^ = 0%). In our overall analysis of the 5 selected RCTs, the fixed effects model showed that patients who received treatment that included TZD therapy experienced a significantly greater reduction in fibrosis than that in their corresponding placebo and control groups (OR: 1.39; 95% CI: 1.01–1.90; *P* = 0.04; Fig. 1A). However, in our subgroup analysis, we found that patients who underwent both TZD therapy and lifestyle changes did not experience significantly greater improvement in fibrosis than that in patients who implemented lifestyle changes alone or lifestyle changes with placebo (OR: 1.55; 95% CI: 0.76–3.17; *P* = 0.23; Fig. 1B). In the subgroup analysis of patients treated without lifestyle changes, we found that improvement in fibrosis in patients who received TZD treatment alone was not significantly greater than that in patients that received a placebo (OR: 1.35; 95% CI: 0.95–1.92; *P* = 0.09; Fig. 1C).

### 3.4 Lobular inflammation reduced by TZD treatment

**Figure 2:**
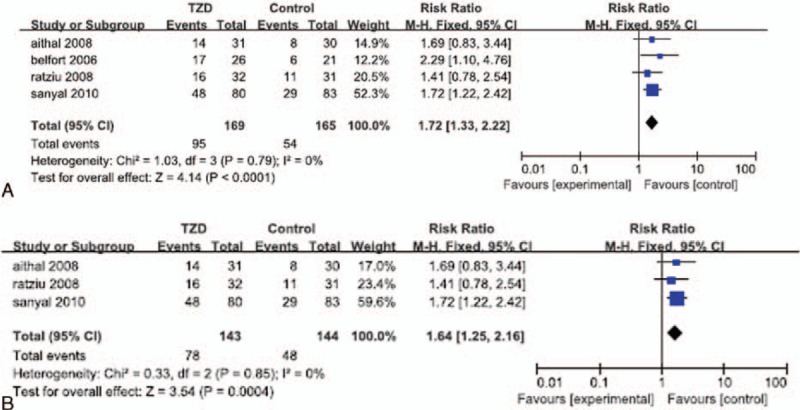
Improvement in lobular inflammation in patients treated with TZDs. (A) Overall analysis of improvement in lobular inflammation. (B) Subgroup analysis of improvement in lobular inflammation in patients treated with TZDs alone.

Data regarding lobular inflammation were reported in 4 of the RCTs.^[23,28–30]^ One study was excluded from the meta-analysis of lobular inflammation because it reported the median inflammation score, whereas the 4 RCTs included in our meta-analysis reported the mean score. Significant heterogeneity in the data regarding lobular inflammation was not detected (*I*^*2*^ = 0%). In the overall analysis of lobular inflammation in 4 of the selected RCTs, the fixed effects model showed that patients treated with a TZD experienced a significantly greater reduction in lobular inflammation, than that in the corresponding placebo groups (OR: 1.72; 95% CI: 1.33–2.22; *P* < 0.0001; Fig. 2A). The subgroup analysis showed that patients who received TZD treatment alone had significantly greater improvement in lobular inflammation than that in patients who received a placebo (OR: 1.64; 95% CI: 1.25–2.16; *P* = 0.0004; Fig. 2B).

### 3.5 Lack of IR improvement in TZD-treated patients

**Figure 3:**
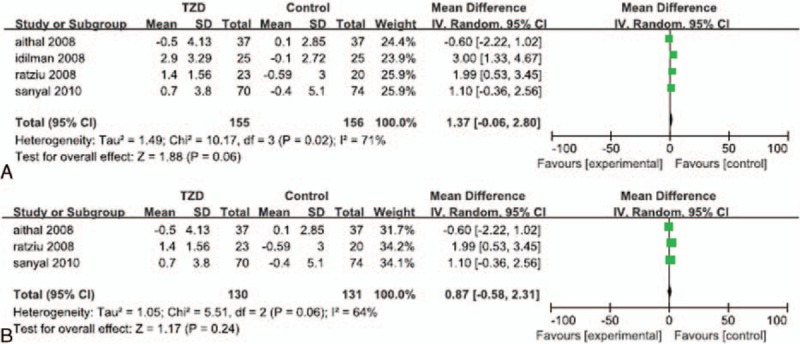
Reduction of HOMA-IR in patients treated with TZDs. (A) Overall analysis of reduction in HOMA-IR. (B) Subgroup analysis of reduction of HOMA-IR in patients treated with TZDs alone.

Data regarding HOMA-IR were reported in 4 of the RCTs.^[24,28–30]^ Significant heterogeneity was detected in the HOMA-IR data (*I*^*2*^ = 71%). The random effects model showed no significant difference between the change in HOMA-IR for patients treated with a TZD and that of their corresponding control group (MD: 1.37; 95% CI: -0.06 to 2.80; *P* = 0.06; Fig. 3A). The subgroup analysis also found no significant difference in the change in HOMA-IR of patients treated with a TZD alone, compared with that of the placebo groups (MD: 0.87; 95% CI: -0.58 to 2.31; *P* = 0.24; Fig. 3B).

### 3.6 Fasting insulin level reduced by TZD treatment

**Figure 4:**
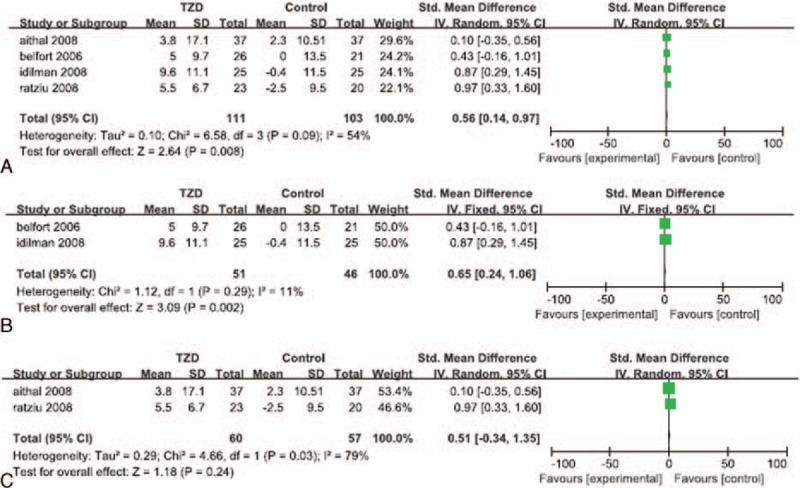
Reduction of fasting insulin level in patients treated with TZDs. (A) Overall analysis of reduction in fasting insulin. (B) Subgroup analysis of reduction in fasting insulin level in patients who underwent lifestyle changes with or without TZD therapy. (C) Subgroup analysis of reduction in fasting insulin level in patients treated with TZDs alone.

Data regarding fasting insulin levels were reported in 4 of the RCTs.^[23,24,29,30]^ Significant heterogeneity was detected in the data reported for fasting insulin levels (*I*^*2*^ = 54%). The random effects model showed that patients treated with a TZD experienced a significantly greater reduction in their fasting insulin level, than that of patients in their corresponding control group (SMD: 0.56; 95% CI: 0.14–0.97; *P* = 0.008; Fig. 4A). The subgroup analyses showed that patients who underwent both TZD therapy and lifestyle changes experienced a significantly greater reduction in their fasting insulin level than that of patients who underwent lifestyle changes alone or lifestyle changes with placebo (SMD: 0.65; 95% CI: 0.24–1.06; *P* = 0.002; Fig. 4B). However, patients treated with TZDs alone did not experience a significantly greater reduction in their fasting insulin level than that of patients who received a placebo (SMD: 0.51; 95% CI: -0.34 to 1.35; *P* = 0.24; Fig. 4C).

### 3.7 Adiposity-related adverse effects of TZD treatment

**Figure 5:**
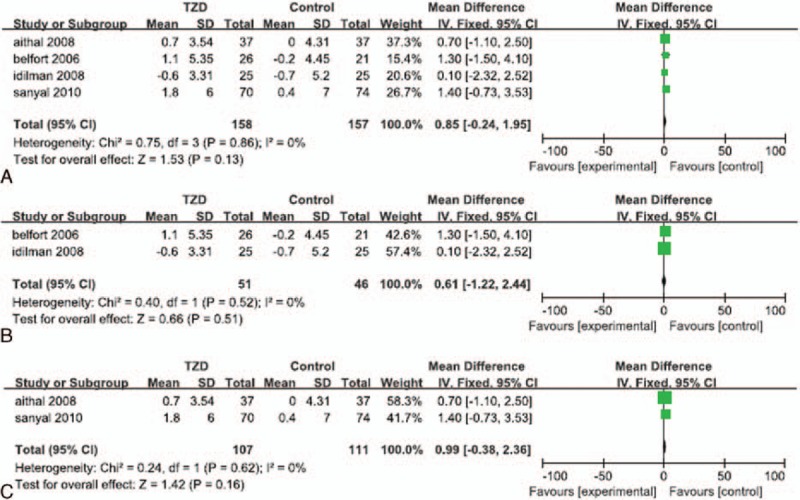
Increase in BMI in patients treated with TZDs. (A) Overall analysis of increase in BMI. (B) Subgroup analysis of BMI increase in patients who underwent lifestyle changes with or without TZD therapy. (C) Subgroup analysis of BMI increase of patients treated with TZDs alone.

Data regarding increases in BMI were reported in 4 of the RCTs.^[23,24,28,30]^ Significant heterogeneity was not detected in the BMI data (*I*^*2*^ = 0%). The fixed effects model showed no significant difference in the change in BMI between the TZD-treated patients and the control patients (MD: 0.85; 95% CI: -0.24 to 1.95, *P* = 0.13; Fig. 5A). The subgroup analyses showed that the increase in BMI in patients who underwent both lifestyle changes and TZD therapy was not significantly greater than that in patients who implemented lifestyle changes without TZD treatment (MD: 0.61; 95% CI: -1.22 to 2.44; *P* = 0.51; Fig. 5B), and that there was no significant difference in the change nimbi in patients treated with TZDs alone, compared with that in the placebo groups (MD: 0.99; 95% CI: -0.38 to 2.36; *P* = 0.16; Fig. 5C).

**Figure 6:**
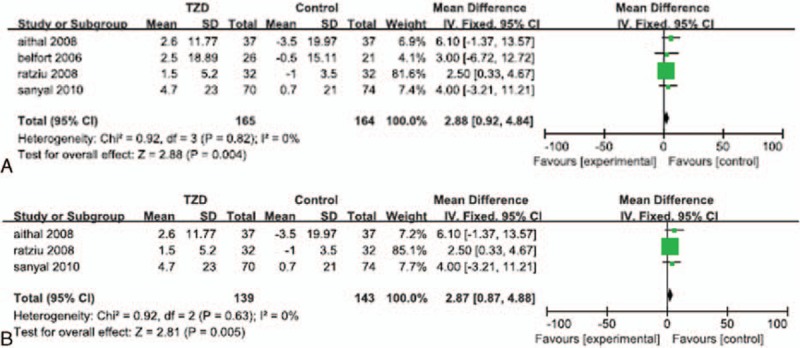
Weight gain in patients treated with TZDs. (A) Overall analysis of weight gain. (B) Subgroup analysis of weight gain in patients treated with TZDs alone.

Weight gain was reported among the patients treated with TZDs in 4 of the RCTs.^[23,28–30]^ Significant heterogeneity was not detected in the weight gain data (*I*^*2*^ = 0%). The fixed effects model showed a significantly greater increase in body weight occurred among the TZD-treated patients, than that in the control groups (MD: 2.88; 95% CI: 0.92–4.84; *P* = 0.004; Fig. 6A), and the subgroup analysis showed that patients treated with TZDs alone experienced a significantly greater increase in body weight than that in the placebo groups (MD: 2.87; 95% CI: 0.87–4.88; *P* = 0.005; Fig. 6B).

**Figure 7:**
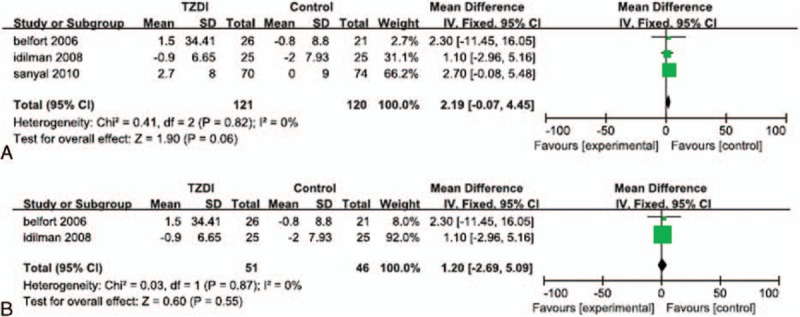
Increase in percentage of body fat in patients treated with TZDs. (A) Overall analysis of increase in body fat percentage. (B) Subgroup analysis of increase in body fat in patients who underwent lifestyle changes with or without TZD therapy.

Increases in body fat were reported among the patients treated with TZDs in 3 of the RCTs.^[23,24,28]^ Significant heterogeneity was not detected in the body fat percentage data (*I*^*2*^ = 0%). The fixed effects model showed that the increase in the percentage of body fat among the TZD-treated patients was not significantly greater than that in the control or placebo groups (MD: 2.19; 95% CI: -0.07 to 4.45; *P* = 0.06; Fig. 7A). The subgroup analysis also showed that the increase in percentage of body fat among patients who underwent both lifestyle changes and TZD therapy was not significantly different than that in the patients who implemented lifestyle changes without TZD treatment (MD: 1.20; 95% CI: -2.69 to 5.09; *P* = 0.55; Fig. 7B).

### 3.8 Dyslipidemia-related adverse effects of TZD treatment

**Figure 8:**
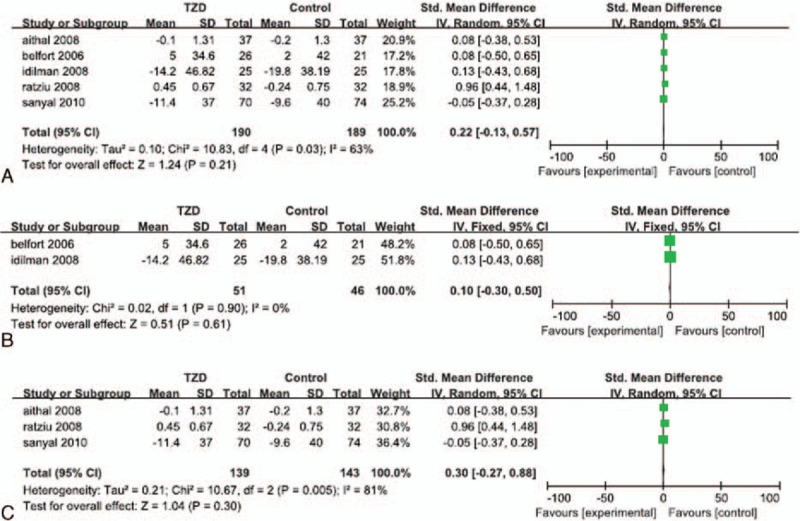
Increase in serum cholesterol level in patients treated with TZDs. (A) Overall analysis of increase in serum cholesterol. (B) Subgroup analysis of increase in serum cholesterol in patients who underwent lifestyle changes with or without TZD therapy. (C) Subgroup analysis of increase in serum cholesterol in patients treated with TZDs alone.

Increases in the serum cholesterol level were reported in all of the 5 RCTs. Significant heterogeneity was detected in the serum cholesterol data (*I*^*2*^ = 63%). The random effects model showed that the increase in the level of serum cholesterol in TZD-treated patients was not significantly different than the change in serum cholesterol in the corresponding control patients (SMD: 0.22; 95% CI: -0.13 to .57; *P* = 0.21; Fig. 8A). The subgroup analyses showed that the change in serum cholesterol in patients who underwent both lifestyle changes and TZD therapy was not significantly different than that in patients who implemented lifestyle changes with or without placebo (SMD: 0.10; 95% CI: -0.30 to 0.50; *P* = 0.61; Fig. 8B), and that the change in serum cholesterol in patients treated with TZDs alone was not significantly different than that in the placebo groups (SMD: 0.30; 95% CI: -0.27 to 0.88; *P* = 0.30; Fig. 8C).

**Figure 9:**
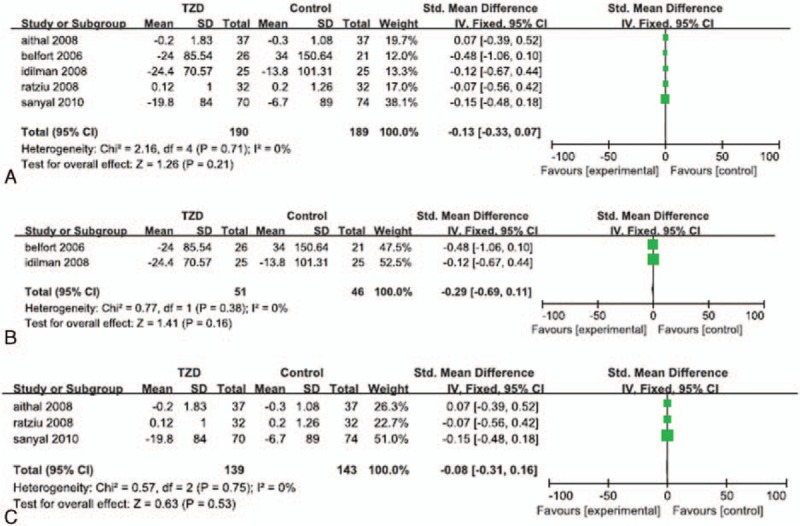
Increase in serum triglyceride level in patients treated with TZDs. (A) Overall analysis of increase in serum triglyceride. (B) Subgroup analysis of increase in serum triglyceride in patients who underwent lifestyle changes with or without TZD therapy. (C) Subgroup analysis of increase in serum triglyceride in patients treated with TZDs alone.

Increases in the level of serum triglyceride were reported in all of the 5 RCTs. Significant heterogeneity was not detected in the serum triglyceride data (*I*^*2*^ = 0%). The fixed effects model showed that the change in the serum triglyceride level in TZD-treated patients was not significantly different than that in the corresponding control groups (SMD: -0.13; 95% CI: -0.33 to 0.7; *P* = 0.21; Fig. 9A). The subgroup analyses showed that the change in serum triglyceride in patients who underwent both lifestyle changes and TZD therapy was not significantly different than that in patients who implemented lifestyle changes with or without placebo (SMD: -0.29; 95% CI: -0.69 to 0.11; *P* = 0.16; Fig. 9B), and that the change in serum triglyceride in patients treated with TZDs alone was not significantly different than that in the placebo groups (SMD: -0.08; 95% CI: -0.31 to 0.16; *P* = 0.53; Fig. 9C).

## 4 Discussion

Although previous studies have suggested that NASH patients treated with TZDs experience improvement in steatosis, lobular inflammation, and hepatocellular ballooning, findings regarding the effects of TZD treatment on hepatic fibrosis have been inconsistent.^[23,29,31]^ Various TZD regimens have also been shown to improve insulin sensitivity in NASH patients,^[14,20–22]^ but the effects of TZDs on important hyperglycemia and adiposity-related variables have not comprehensively assessed across RCTs. In addition, although diet control and long-term exercise have also been shown to improve IR in NASH patients,^[22,32]^ meta-analyses and systematic reviews have not fully assessed the effects of the combination of TZDs and lifestyle changes on histological parameters, serum lipid levels, adipogenesis, and glycemic control in subgroup analyses.^[25,33]^

Our overall analysis of the 5 RCTs included in our study showed that TZD-treated patients experienced improvement in hepatic fibrosis. However, the subgroup analyses showed that a significant improvement in fibrosis did not occur among the patients treated with TZDs alone or in those who underwent both lifestyle changes and TZD therapy. The findings of our overall analysis are consistent with those of a previous systematic review and meta-analysis that used random effects models to evaluate treatment effects in the same 5 RCTs that were included in our study, but did not perform a subgroup analysis to distinguish between the effects of TZDs on fibrosis from those of lifestyle changes in studies in which exercise and/or diet control were combined with TZD therapy.^[25]^ By contrast, the results of our subgroup analyses, which found that TZD treatment did not stem the development of fibrosis in NASH patients, are consistent with those of 2 previous studies, one of which was a recent network meta-analysis.^[33,34]^ Our findings combined with those of previous meta-analyses emphasize the importance of avoiding the overinterpretation of data regarding the effects of TZDs on fibrosis in NASH patients who were subjected to different treatment regimens.

We also examined the change in lobular inflammation as an additional histological marker of NASH progression. Both the overall and subgroup analyses showed that lobular inflammation decreased in NASH patients who received TZD treatment and those who underwent both TZD therapy and lifestyle changes, respectively. Our results were similar to those of 2 previous meta-analyses of 4 of the 5 RCTs included in our study suggesting the need for assessing multiple histological markers of NASH pathogenesis in evaluations of drug therapy in clinical trials. In general, inflammation and fibrosis are representative of mild and severe disease, respectively, and necroinflammatory activity may even be absent in some patients with cirrhosis.^[19]^ Therefore, our findings combined with those of previous studies suggest that, given the lack of clearly defined significant improvement in fibrosis among TZD-treated patients, the benefits of TZD treatment may be more beneficial for patients in the early stages of NASH.

The development of IR contributes to the progression of fatty liver degeneration from simple steatosis to NASH because it stimulates de novo lipogenesis in hepatocytes, and is associated with the development of fibrosis.^[35–37]^ A previous meta-analysis of TZD treatment for NASH showed that an increase in insulin sensitivity was limited to NASH patients with type 2 diabetes mellitus (T2DM).^[25]^ Our overall and subgroup analyses of changes in HOMA-IR found no significant improvement in IR in any of the TZD treatment groups. However, our subgroup analyses showed that NASH patients who underwent both lifestyle changes and TZD therapy experienced a significantly greater reduction in fasting insulin level than that in the control patients, whereas patients treated with TZDs alone did not experience a significantly greater reduction in fasting insulin level than that in patients who received a placebo. These results indicate that additional markers of IR should be analyzed in RCTs of TZD treatments for NASH, and warrant future investigations of the effects of diet control, exercise, and TZD combination therapy on IR in nondiabetic NASH patients to determine whether such treatment regimens can reduce the risk of T2DM. Our findings also suggest that the inclusion of additional glucose-lowering medications in NASH treatment regimens may be beneficial for reducing the risk and severity of IR.

To assess the safety of TZD therapy, we also analyzed the incidence of adverse effects of TZD treatment that are known to be risk factors for T2DM, CVD, and MetS. Although patients treated with TZDs experienced significantly greater weight gain than that which occurred in the control patients, they did not experience a significantly greater increase in BMI or percentage of body fat. Therefore, the underlying mechanism of the weight gain observed was unlikely to involve an increase in adipogenesis. Although both rosiglitazone and pioglitazone are peroxisome-proliferator activated receptor γ (PPARγ) agonists, pioglitazone has been shown to be more effective than rosiglitazone for managing low-density lipoprotein cholesterol and triglyceride levels,^[38,39]^ which may be the result of partial activation of PPARα by pioglitazone.^[40]^ However, we found that TZD therapy had no significant impact on the serum levels of total cholesterol and triglyceride. Previous studies have shown that TZD can increase the serum level of adiponectin, which is known to be associated with an increased risk of MetS and CVD.^[41]^ Our results suggest the need for additional assessments of risk factors for T2DM, CVD, and MetS in clinical studies of TZD treatment for NAFLD or NASH.

In conclusion, our meta-analysis of RCTs of TZD therapy for NASH revealed no significant improvement in liver fibrosis or IR. However, our analysis of fasting insulin levels indicated that additional clinical variables should be assessed to obtain a more comprehensive evaluation of the effects of TZD treatment on insulin sensitivity in NASH patients, and suggested that including diet control, exercise, and additional insulin-sensitizing drugs in TZD treatment regimens may be beneficial for reducing IR in NASH patients. Our analyses of adiposity and dyslipidemia-related adverse effects indicated that additional clinical variables and serum biochemical markers should be assessed in TZD-treated NASH patients to identify the mechanism through which TZDs contribute to weight gain, and to determine whether TZD-induced weight gain is clinically relevant to the risk of T2DM, CVD, and MetS in NASH patients. The relatively small numbers of studies included in our meta-analysis and subgroup analyses reduce the statistical power of our investigation, and thereby serve to limit the interpretation of our findings.
